# Physical, Thermal, and Chemical Properties of Fly Ash Cenospheres Obtained from Different Sources

**DOI:** 10.3390/ma16052035

**Published:** 2023-03-01

**Authors:** Andrei Shishkin, Vitalijs Abramovskis, Ilmars Zalite, Ashish Kumar Singh, Gundars Mezinskis, Vladimir Popov, Jurijs Ozolins

**Affiliations:** 1 Rudolfs Cimdins Riga Biomaterials Innovations and Development Centre of RTU, Institute of General Chemical Engineering, Faculty of Materials Science and Applied Chemistry, Riga Technical University, Pulka 3, K-3, LV-1007 Riga, Latvia; 2 Institute of Materials and Surface Technologies of the Riga Technical University, P. Valdena iela 7, LV-1048 Riga, Latvia; 3 Department of Materials and Engineering, Tel Aviv University, Tel Aviv 6997801, Israel

**Keywords:** hollow microballoons, cenospheres, ceramics phase composition, chemical composition

## Abstract

Cenospheres are hollow particles in fly ash, a by-product of coal burning, and are widely used as a reinforcement when developing low-density composites called syntactic foams. This study has investigated the physical, chemical, and thermal properties of cenospheres obtained from three different sources, designated as CS1, CS2, and CS3, for the development of syntactic foams. Cenospheres with particle sizes ranging from 40 to 500 μm were studied. Different particle distribution by size was observed, and the most uniform distribution of CS particles was in the case of CS2: above 74% with dimensions from 100 to 150 μm. The CS bulk had a similar density for all samples and amounted to around 0.4 g·cm^−3^, with a particle shell material density of 2.1 g·cm^−3^. Post-heat-treatment samples showed the development of a SiO_2_ phase in the cenospheres, which was not present in the as-received product. CS3 had the highest quantity of Si compared to the other two, showing the difference in source quality. Energy-dispersive X-ray spectrometry and a chemical analysis of the CS revealed that the main components of the studied CS were SiO_2_ and Al_2_O_3._ In the case of CS1 and CS2, the sum of these components was on average from 93 to 95%. In the case of CS3, the sum of SiO_2_ and Al_2_O_3_ did not exceed 86%, and Fe_2_O_3_ and K_2_O were present in appreciable quantities in CS3. Cenospheres CS1 and CS2 did not sinter during heat treatment up to 1200 °C, while sample CS3 was already subjected to sintering at 1100 °C because of the presence of a quartz phase, Fe_2_O_3_ and K_2_O. For the application of a metallic layer and subsequent consolidation via spark plasma sintering, CS2 can be deemed the most physically, thermally, and chemically suitable.

## 1. Introduction

In recent years, syntactic foam (SF) composites have increasingly found use in various industries where superior mechanical strength at a low weight is desired. SF is made of polymer or metal in between hollow microspheres [1,2,3]. Syntactic foam with specific properties can be obtained by reinforcing hollow particles in a polymer, metallic, or ceramic matrix. Syntactic foams are increasingly being used for their excellent mechanical strength and stiffness-to-weight ratios [4,5] Such materials are used in aviation, space technology, and ship construction to manufacture individual parts [6,7].

A promising material for obtaining syntactic foam is cenospheres (CS), hollow aluminosilicate microballoons. CS is a waste product in the composition of fly-ash burning coal in thermal power plants (TPC), as a result of complex thermochemical processes [8]. Recently, coal thermal power plants have produced about 41% of global electricity, which is expected to increase to about 44% by 2030 [8]. This combustion of coal results in the production of large quantities of waste called fly ash. It is estimated that approximately 750 million tonnes of fly ash are produced annually worldwide, and this is increasing at a high rate. A large quantity of fly ash is disposed of in landfills and ash lagoons. Due to the heavy metal leaching and high cost of landfills, many applications have been suggested to convert this waste into value-added products. To increase the re-use of coal fly ash, several separation methods have been developed to segregate value-added components, such as aluminosilicates, magnetite, and CS from solid particles and unburned carbon [8]. CS can be separated from the ash by the method of flotation; in lagoons, they float to the surface of the water, from where they can be collected for further processing. The CS world market is worth up to USD 600 million annually, equivalent to 700–800 tonnes [9,10]. The main suppliers of CS in the world are China, India, Russia, Kazakhstan, and Ukraine. As a waste product of TPC operations, CS are available at a relatively low cost.

Cenospheres account for an average of 0.01 to 4.80% by mass of fly ash. They are characterised by a low bulk density (0.4–0.72 g·cm^−3^), very low thermal conductivity (about 0.065 W·m^−1^·K^−1^), and excellent stability in an alkaline solution and up to high temperatures. The particle size of CS ranges from about 5 to 500 µm [8,11]. By chemical composition, these materials are multi-component systems with a SiO_2_–Al_2_O_3_–Fe_2_O_3_ content of approximately 90 wt% [12,13,14]. The utilisation of fly ash is determined by its properties, such as fineness, specific surface area, particle shape, hardness and freeze–thaw resistance [15,16].

The properties of CS and their significant accumulations can be recycled long-term and used to create new functional materials with a high added value. CS are very widely used in the manufacture of stuffed building materials with a reduced density and improved thermal insulation properties [9,10] and are also used as a filler and reinforcement in metal–matrix composites [17,18,19] ceramics–matrix composites [20,21,22], polymer–matrix composites [23,24] and various hybrid matrix composites [21,25]. Cenospheres can be employed with polymers and hybrid matrices intended for low-temperature applications [26,27,28,29] as well as for the development of geopolymer composites [20,30,31]. In both cases of material production, the working temperatures do not exceed 250 °C; therefore, these studies have not paid significant attention to the effect of temperature on the physical properties or CS stability. High-temperature cases for the CS have also been studied. Materials intended for applications such as fire protection [30,32] and thermal barriers [32] have employed CS, and typically experience temperatures above 900 °C. Other high-temperature conditions exist for CS, such as the fabrication of metal–matrix composites [33,34,35,36], where relatively high (up to 900–1200 °C) temperatures can be seen. However, these studies too lack the much-desired understanding of the changes in CS properties during the thermal treatment.

A promising direction for their use may be to manufacture lightweight and mechanically resistant multi-layered composite materials with specific properties. Previous work shows a proof-of-the-concept of obtaining novel (matrix-less) SF [37,38] by using CS from burnt coal from the Donetsk and Ekibastuz coal basins’ TPC. As described in works [37,38], matrix-less SFs were obtained by the spark plasma sintering (SPS) of metallised CS (Me@CS) at a temperature range from 800 °C (for Cu) up to 1150 °C in the case of Ti and 316 steel coatings. A micron and a sub-micron layer of metal (Cu, Ti, and 316 steel) [37,38] were deposited via physical vapour deposition (PVD).

The development of SF from metallised CS has been studied previously and by other researchers. However, neither the effect of the heat treatment and processing on the properties of CS, nor the difference between the behaviour of CS obtained from different sources is well understood. It is especially important to understand this, given the prevailing geopolitical situation, which is making it challenging to source raw materials with the same ease and availability. Since the properties of CS depend on the type of coal and the conditions of a TPC, CS have a wide range of properties [8,11]. The authors believe that it is necessary to understand the influence of temperature on the structure, physical, and chemical properties in the heat treatment of CS available from different thermal power plants.

The purpose of this work is to understand the quality of the raw material, the effect of heat treatment on the CS, and their characterisation, which could be suitable for the preparation of novel matrix-less SF precursors. In this research, the phase change that occurs in the CS during the pre-treatment will be studied. The physical and chemical properties of raw CS and metallised CS are studied to better inform and guide subsequent processes, i.e., the development of SF and its mechanical properties.

## 2. Materials and Methods

The cenospheres used in the current study were a by-product of coal-fired plants from the Donetsk and Ekibastuz coal basins, burning them in various TPC. The origin, designation, and particle size characteristics of CS are shown in Table 1.

A determination of the particle size distribution (PSD) was carried out with the help of the vibratory sieve shaker Analysette 3 PRO (FRITSCH GmbH, Idar-Oberstein, Germany). Particles were fractioned using sieves with opening sizes of 0.05, 0.10, 0.15, 0.20, 0.25, and 0.30 mm. The bulk density was determined by using a bulk density tester (Scott volumeter, according to ASTM B 329-98, Copley, Nottingham, UK). To obtain the true particle density (TPD), a Quantachrome Ultrapyc 1200e (Anton Paar GmbH, Graz, Austria) automatic gas pycnometer was used.

Specific thermal conductivity was determined using LaserComp (ASV) Fox 600 measuring equipment, and the sample was freely poured into a layer with a thickness of 30 mm.

### 2.1. Determination of the Interparticle Void Fraction

Before producing the syntactic foams, it is essential to know the maximum achievable packing density or its opposite value—interparticle void fraction *ɛ*. This value gives the maximum volume fraction (VF) of CS that can be dispersed in the matrix. The maximum possible packing density for spherical particles is known to be 0.741 [39,40]. In the case of practically monodispersed particles, with possible dense packing (random close packing), this value can reach 0.64 [41,42,43,44], while in the case of real loose packing (random loose packing), it usually does not exceed 0.60 [41]. This means that an actual interparticle void fraction is not less than *ɛ* = 1–0.60 = 0.40. The determination of the interparticle void or volume filling coefficient *ε* was calculated using Equation (1):

(1) $$\varepsilon =\frac{V-\left(V_{p+fl}-V_{fl}\right)}{V}$$

where:

*V*—the volume of the particles in bulk condition, prepared with Scott volumeter,
$V_{p+fl}$—the volume of the mixture (particles + fluid),
$V_{fl}$—the volume of the fluid.
*A*—method for determining the CS interparticle voids was developed.

The essence of this is as follows. The volume of the particles in the bulk condition (*V*) is the sum of the volume occupied by the particles and the interparticle void volume, which can be determined by filling the voids with a certain volume of fluid *V_fl_*. Since the bulk density of CS is usually in the range of 0.38 to 0.43 g·cm^−3^, it is impossible to perform the experiment correctly using water. The forces acting on a dispersed particle in a viscous medium are described by Stokes’s law, which states that the sedimentation rate of a particle under other independent conditions is proportional to the difference between the densities of the particle and the medium, and inversely proportional to the viscosity of the medium. The kinetic persistence of dispersed systems (or, more simply, the sedimentation speed of particles or the particle rise speed) is described by Stokes’s law, Equation (2):

(2) $$\;V_{S}=\frac{d^{2}g\left(\rho_{p}-\rho_{fl}\right)}{18\;\mu }$$

where $V_{S}$ is the sedimentation speed of particles in m·s^−1^ (if $\rho_{p}>\rho_{fl}$) or the particle rise speed to the surface of a liquid (if $\rho_{p}<\rho_{fl}$).

*d*—diameter of the particle (m),
*g*—acceleration of gravity (m·s^−^²),
$\rho_{p}$—single particle apparent density (kg·m^−^³),
$\rho_{\mathrm{fl}}$—density of the fluid (kg·m^−^³),
*μ*—dynamic viscosity of the fluid (Pa·s).

Based on Stokes’s Formula (2), the sedimentation (stratification) speed is directly proportional to the particle diameter, the phase difference, and the square of the density of the environment, as well as inversely proportional to the viscosity of the environment. Therefore, to reduce the sedimentation rate, i.e., to increase the resistance to sedimentation, different methods can be used, such as increasing the viscosity of the environment using dispersants with a density close to the density of the substance.

The simplest way is to increase the viscosity of the environment dispersion. To control the viscosity, a solution of potato starch in distilled water (10 g·L^−1^), with 0.01% (−)-Ethyl L-lactate (purists. p.a., cleaning grade, ≥98.0% (sum of enantiomers, GC), supplier—Fluka (now Sigma-Aldrich)) as surfactant, was used. The exact volume of CS (25 cm^3^) under investigation was obtained with a Labulk-0302 Scott Volumeter (Version ISO 3923-2) to ensure the actual bulk density, and then 25 cm^3^ of CS was mixed with a precise (100.0 cm^3^) volume of starch solution. The system was then degassed, and a precise volume measurement was taken.

### 2.2. Determination of the Ratio between Intact and Defective Cenospheres

To obtain the highest quality syntactic foam from metallised CS, separating the intact CS from the damaged ones is necessary. The damaged CS can be: broken, porous and/or cracked from handling or processing. For this, the flotation method was chosen and implemented as follows. A solution consisting of 800 cm^3^ of distilled water and 50 cm^3^ of ethanol (to reduce surface tension) was prepared, and 100 g of CA was added and stirred with a glass rod for 60 s. The mixture was sifted for 5 min, and then the floating fraction (with the aid of a spatula and decantation) was carried to the next beaker with fresh washing liquid, and the procedure was repeated. After repeated settling of the upper layer, it was transferred to a Büchner funnel and washed with ethanol. Then, it was dried in an oven at 105 °C for 12 h with a layer of no more than 20 mm. To determine the ratio of intact and destroyed CS, the liquid collected below settlings was decanted carefully and the sediments were transferred to a Büchner funnel. It was then rinsed with distilled water, and then by ethanol, and finally dried in an oven at 105 °C for 12 h.

### 2.3. Morphology, Chemical and Phase Composition

A Scanning Electron Microscope (SEM) Zeiss EVO MA-15 EDS (used at 5 kV) equipped with INCA Energy 350 energy-dispersive X-ray spectroscopy (EDS) at 15 kV was used to evaluate the morphology and element composition of the CS. For the elemental composition via EDS, 10 points were used—from the surface and from the inside (fractured CS) of the cenospheres. For the phase-composition determination, powder X-ray diffraction (XRD) on a BRUKER D8 Advance diffractometer with a Cu anode, accelerating voltage of 40 kV, and beam current of 40 mA, with 0.02° per step, was applied. For quantitative analysis, the Rietveld method with internal standard mineral corundum was used.

A high-temperature optical microscope (HTM) (EM201 HT163, Hesse instruments, Germany) was used for 2D-dilatometry, which is an optical dilatometry—taking cross-section measurements of the specimen’s area. A cylindrical shape (d = 5 and h = 7 mm) was used for the test. The heating mode was set as follows: up to 800 °C, the heating rate was 20 °C·min^−1^, since up to this temperature, deformations of the CS are unlikely to occur; however, heating above 800 °C was carried out at a speed of 5 °C·min^−1^.

Fourier transform infrared (FTIR) spectra were recorded using a Thermo Scientific Nicolet™ iSTM50 (Thermo Fisher) spectrometer in the Attenuated Total Reflectance (ATR) mode. Spectra were obtained over a range of wavenumbers from 400 cm^−1^ to 4000 cm^−1^, co-adding 64 scans at a 4 cm^−1^ resolution. Before every measurement, a background spectrum was taken and deducted from the sample spectrum.

Loss of ignition (LOI) was determined at temperatures of 400 and 1000 °C, according to GOST 2642.2-86. The chemical analysis was performed according to GOST 32362-2013.

### 2.4. Material Preparation and Study

It is necessary to understand the thermal–structural relationship for CS in order to obtain advanced SF, since the method of foam synthesis is SPS. The sintering of metallised CS involves the melting and fusion of the metal coating that can reach temperatures in the range of 1100–1200 °C [37,38]. The composition of CS is refractory ceramics that can withstand high temperatures, but it is necessary to investigate the effects of this thermal treatment on the CS.

**Figure 1:**
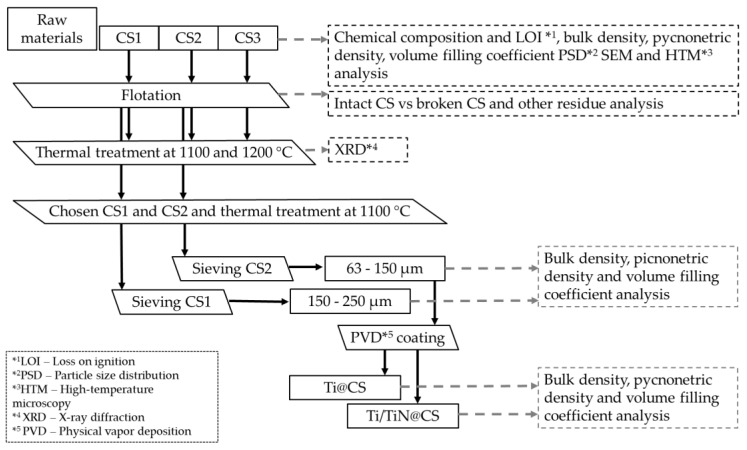
Flowchart depicting the process flow and characterization after each step.

The process flowchart for the study is shown in Figure 1, which describes the treatment and characterization steps taken for cenospheres CS1, CS2 and CS3. The bulk density (ρ_bk_), pycnometric density(ρ_py_), interparticle void fraction (ε), particle size distribution (PSD), LOI, SEM, phase composition and 2D-dilatometry (HTM analysis) were determined. To ensure the high quality of the metal–CS foam by sintering of the metallised CS, it is necessary to separate the intact and damaged CS. For this purpose, the flotation method was used with a liquid medium—in this case, water. After the separation of damaged CS and unburned coal particles, the influence of 1100 and 1200 °C thermal treatment on the CS was studied. The thermal treatment was performed in a Nabertherm Laboratory Furnace L9/13 with a P330 controller (Nabertherm, Lilienthal, Germany), using an open alumina crucible (V = 300 cm^3^), filled to 2/3 of its total volume, in an air atmosphere, with a heating rate of 5 °C·min^−1^ and dwell time of 30 min with subsequent natural cooling.

## 3. Results

### 3.1. Granulometry Composition and Density of CS

**Figure 2:**
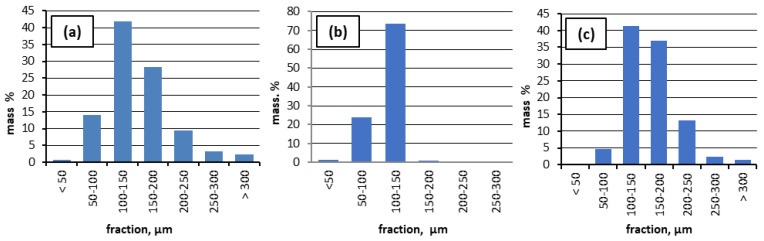
Particle size distribution (PSD) of the three types of cenospheres: (**a**) CS1, (**b**) CS2, and (**c**) CS3.

For all types of CS, the particle size distribution was determined. As can be seen from Figure 2, the CS used in the experiments are predominantly (more than 40%) in the 100 to 150 μm size range. In the case of CS1, up to 14% are particles of <100 μm, and about 27% are particles of >150 μm. In the case of CS2, particles with average sizes of 100–150 μm are mainly present, while in CS3, particles (up to 42%) with sizes of 100–150 μm and particles of >200 μm (up to 15%) are observed. From a granulometric point of view, a more even distribution of CS particles is seen in the case of CS2: above 74% with dimensions from 100 to 150 μm.

Important indicators are the bulk density of the material, the interparticle void fraction, as well as the density of the material of the globular wall. The experimental data are summarised in Table 2. The lowest void fraction (38.0 %) corresponds to the most polydispersed specimen—CS3. Additionally, the highest void fraction (46.0 %) corresponds to CS1 after sieving and flotation (specimen CS1, 150–250 µm, treated at 1100 °C). The lowest bulk density is observed in the case of CS2, which may be due to a more even particle size distribution, as was seen in Figure 2. Dispersions with different particle sizes during pouring are arranged more densely, which leads to an increase in bulk density and a decrease in the interparticle void volume. The pycnometric density after heat treatment at 1100 °C increased from 2.153 to 2.178 g·cm^−3^ in the case of the CS1 raw material. In the case of CS1, the pycnometric density mainly corresponds to the CS wall material, namely ceramics, and the density could change due to a phase composition change. In the case of CS2, the pycnometric density decreased from 2.272 to 2.185 g·cm^−3^. This fact could be explained by the burning out of residual coal particles (C content up to 11.5 at.% and LOI = 0.4) during the thermal treatment (Table 3 and Table 4), and the significant amount of broken CS (11.6%) content in the CS2 sample, which is separated later by the flotation method to prepare the CS 63–150 µm fraction. Ti- and Ti-TiN-coated CS have a greater pycnometric density, which is obvious, due to the deposited Ti and Ti-TiN.

### 3.2. Chemical Composition

In order to more fully characterise the microspheres studied, it is important to find out and compare their chemical composition, which can differ significantly when burning different coals at different temperatures. Energy-dispersive X-ray spectroscopy data from the surface of the raw microspheres show that the main elements present in their shell are Al, Si, Fe, Ca, Mg, K, Na, O, and in significant quantities, C. The average compositions of the microsphere material elements from EDS analysis data are shown in Table 3.

It should be noted that the composition of the material is rather uneven for all samples. Samples CS1 and CS2 have the same elemental composition and Si/Al ratio, but sample CS3 is significantly different. The content of Al has practically been increased twofold, and Fe and K appear in the composition of the material in noticeable quantities. For all samples, the elemental analysis shows significant amounts of carbon, which could be explained by the carbon that was not fully burned.

The chemical analysis was performed by converting the analysed elements to oxides. For the LOI determination, the studied samples were thermally treated at temperatures of 400 and 1000 °C (see Table 4).

The results of the chemical analysis confirm the obtained EDS data—CS1 and CS2 are close enough in composition, but CS3 is significantly different. Additionally, the main differences are in the proportions of SiO_2_ and Al_2_O_3_ and in the content of Fe_2_O_3_. It should also be mentioned that the mass loss during heating in the case of sample CS3 is on average seven times higher than in the other samples.

### 3.3. The Ratio between Intact and Defective Cenospheres

**Figure 3:**
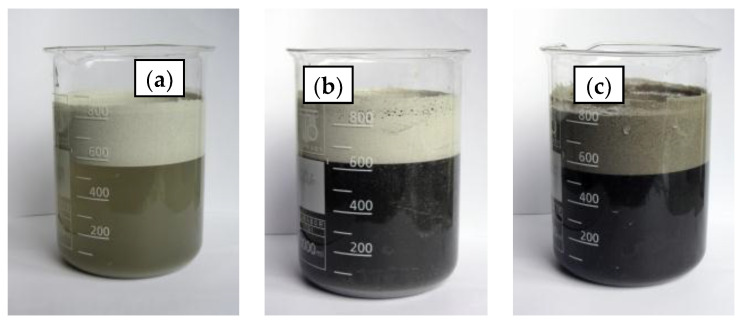
Images of beakers with water and CS1 (**a**), CS2 (**b**), and CS3 (**c**), showing the setup used to separate the broken and intact particles.

When analysing the samples CS1, CS2, and CS3, it was found that after the settling (segregation in floating and non-floating fractions) of samples CS2 and CS3 (Figure 3), the liquid turned black. After filtering, black deposits remained on the filter. Sediment analysis was not carried out, but these are believed to have been formed by particles of coal that had not burned out. In the sample, the CS1 liquid became greyish yellow.

The results of the floating and non-floating microsphere fractions, as well as the calculations of the percentage of the fraction are: 1.1 ± 0.05% defective microspheres in sample CS1, in CS2—11.6 ± 0.09%, in CS3—3.2 ± 0.08%. In the contents of samples CS2 and CS3, it is possible that coal particles that had not burned out were present.

### 3.4. Determination of Crystalline Phases in Cenosphere Composition

**Figure 4:**
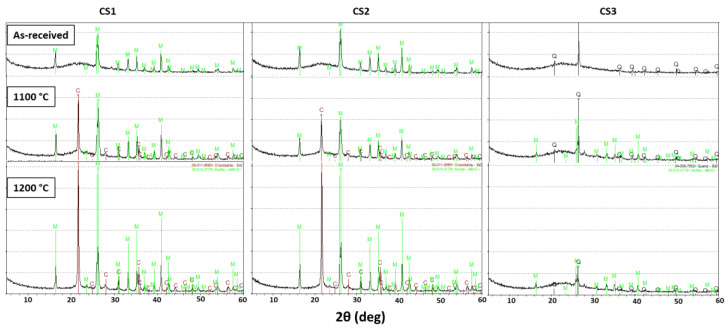
X-ray diffractograms of the CS at as-received and heat-treated conditions. Phase designations: C—cristobalite SiO_2_; Q—quartz SiO_2_; M—mullite Al_6_Si_2_O_13_.

To determine the phase composition of the CS and how it is affected by heat treatment, X-ray diffraction (XRD) phase analysis was performed on as-received and heat-treated CS. The heat treatment of the CS was performed at 1100 and 1200 °C in accordance with the process flow shown in Figure 1. Cenospheres form a small fraction of the fly ash that is produced as a by-product of the mineral component of coal when they burn at high temperatures, with the formation of crystallite mixtures and a glass phase. The diffractograms for CS1, CS2, and CS3 are shown in Figure 4. As shown by the X-ray studies, the diffractograms of samples CS1 and CS2 are very lax. In the output samples, mainly mullite Al_6_Si_2_O_13_ and the amorphous phase, which can be attributed to the glass phase, can be observed. Mullite is the main crystalline phase, which occurs as a result of thermochemical transformations of the aluminosilicates contained in coal. When the samples are heated at 1100 °C and 1200 °C, the phase of cristobalite SiO_2_ is shown, and its intensity increases with the increasing heat-treatment temperature (sample diffractograms are shown in Figure 4). The cristobalite (SiO_2_) phase is not seen in the as-received samples from all sources, but upon heat treatment, it is seen that the amount of the cristobalite phase increases, showing that heat treatment leads to the formation of cristobalite. Along with that, the net amount of the mullite phase also increases, indicated by the increase in peak height.

Sample CS3 differs from the rest in terms of phase composition, showing that it is less crystalline than CS1 and CS2. Additionally, SiO_2_ is already present in the polymorphic modification of quartz in the untreated sample, and mullite forms after heat processing. Moreover, in sample CS3, the amount of the amorphous phase after heat treatment is much higher than in the other two samples (the characteristic plateau on the X-ray diffraction pattern is indicative of this), but in samples CS1 and CS2, it turned into a crystalline phase.

The diffractograms of sample CS3 are sharply different from CS1 and CS2. In the untreated material, it was possible to distinguish the quartz crystallographic phase only, but other phases in an amorphous state (also Al_2_O_3_). In the output sample, only the crystalline phase of quartz at 26.2 degrees at 2Ɵ and the X-ray phase are detected. With a decrease in the concentration of Al_2_O_3_, that is, with an increase in the SiO_2_/Al_2_O_3_ ratio, the quartz phase prevails. The heat treatment of the samples causes the quartz phase to decrease and the formation of the mullite phase.

**Figure 5:**
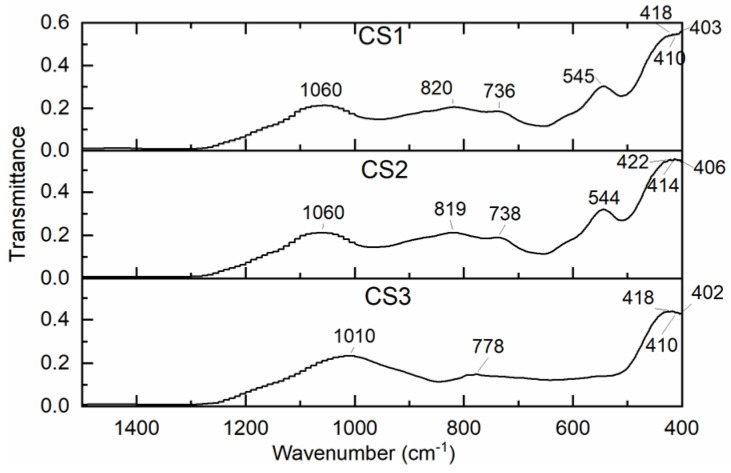
The FT-IR spectra of the cenospheres CS1, CS2, and CS3.

Figure 5 shows that the visible FT-IR spectra of the microspheres studied show a wide absorption band in the interval of 1058–1062 cm^−1^, which can be attributed to the valence and deformation oscillations of Si-O-Si(Al) in the crystal lattice and v = 820 cm^−1^ to valence oscillations in the case of Si-O in CS1 and CS2. In the case of CS3, a pronounced band at 1008 cm^−1^ appears, which is attributed to valence oscillations in the bridge links in the Si-O-Si(Al) crystal lattice. A small maximum also indicates the presence of quartz in the case of CS3 at 778 cm^−1^ [45].

The various valence oscillations of Si-O-Al, Al-O, Si-OH, and Si-O can be attributed to the many absorption bands observed in the interval of 800–420 cm^−1^. The positions of the absorption bands and their intensity in the IR spectra are significantly influenced by the ratio in the structure of Si/Al microspheres. Moreover, this is not about pure materials, but about a technical product with a sufficiently heterogeneous composition.

### 3.5. The Morphology of the CS

**Figure 6:**
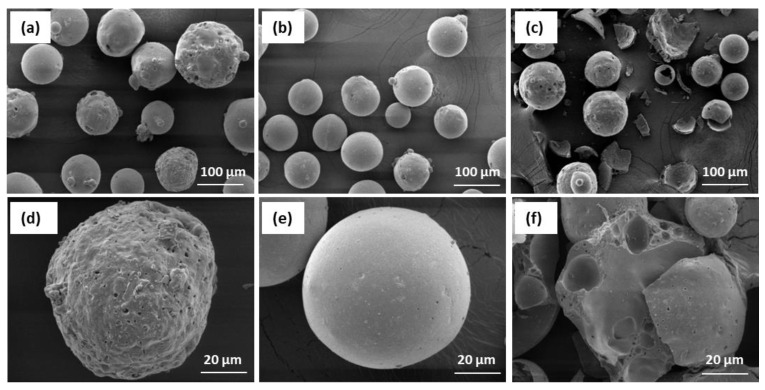
SEM micrographs of the cenospheres: CS1 (**a**,**d**); CS2 (**b**,**e**); CS3 (**c**,**f**).

The chemical and phase composition of CS largely determines the morphology of their surface. Figure 6 shows SEM photomicrographs of the three cenospheres, with all three showing a different surface morphology.

The cenospheres in sample CS1 (Figure 6a) can be seen to have different globular features on the surface, and most of the surface is embossed and perforated. In the case of CS2 (Figure 6b), the particles are of uniform size with high sphericity and a smooth surface. The CS3 cenospheres (Figure 6c) have a wide variety of morphological forms and a significant amount of them are damaged. The walls of the particles have a distinct porosity. The variety of shapes and surface conditions of CS is explained by the very complex processes of transformation of oxide formation and crystallisation after coal burning during the separate stages of their combustion at high temperatures.

**Figure 7:**
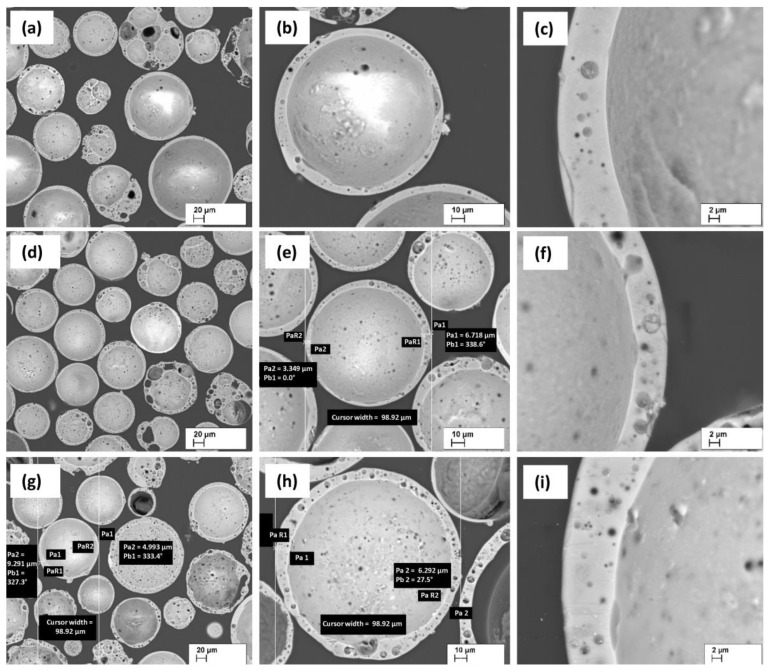
SEM micrographs of CS1 (**a**–**c**), CS2 (**d**–**f**), and CS3 (**g**–**i**) at 200, 500, and 2000 × times magnification, respectively.

In order to be able to judge the thickness of the wall of the CS shells and their structures, resin-fixed CS grindings were made (Figure 7). In most cases, when the CS have a pronounced spherical shape, the shell-wall thickness is from 4 to 10 μm on average. As can be seen, the walls have closely spaced pores, with average pore sizes from 0.5 to 5.0 μm. Additionally, it is clearly seen that CS3 walls have the greatest and closest porosity among all the studied CS.

**Figure 8:**
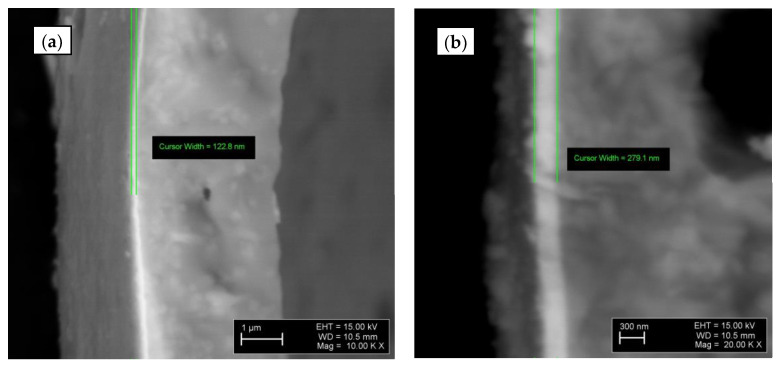
SEM micrographs of the Ti@CS (**a**) and Ti-TiN@CS (**b**).

Figure 8 shows the Ti-deposited layer (122.8 nm) on the CS2 (Figure 8a), and Ti-TiN-deposited layers (279.1 nm) on the CS2 (Figure 8b). However, multiple measurements noted that the layer thickness of Ti (for Ti@CS) is between 100 and 350 nm; and in the case of Ti-TiN@CS, the layer of TiN nitride is between 90 and 350 nm and Ti between 150 and 380 nm. The deposited Ti and Ti-TiN coatings in all the observed specimens are intact and have no cracks, pores, or delamination

### 3.6. The Behaviour of the CS at Elevated Temperature

**Figure 9:**
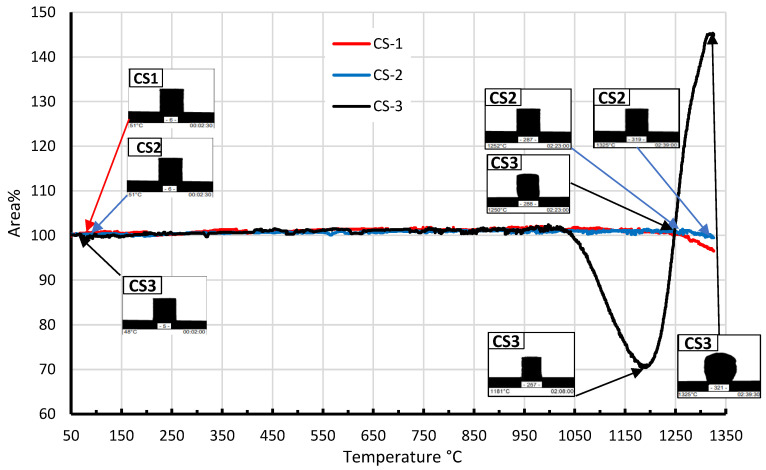
CS1, CS2 and CS3 2D-dilatometry curves in the 50–1325 °C temperature interval.

Using HTM, 2D-dilatometry curves were obtained that describe changes in the volume or linear dimensions of CS under the influence of temperature. As can be seen from the curves in Figure 9, in the patterns made of CS1 and CS2, the deformation is negligible until the 1200–1325 °C temperature interval, but after this temperature, a slight shrinkage can be observed, which could be due to the deformation of the CS and their mutual compaction (softening begins).

As the samples reach 1350 °C, CS1 demonstrates greater (up to 5%) shrinkage than CS2. At the CS3 temperature above 1050 °C, a rapid shrinkage of the sample occurs, which is associated with the transition of the sample to a semi-pyroplastic state. This effect could be caused by the presence of K_2_O and Fe_2_O_3_, resulting in a lower softening point (Tg) of the sample. Additionally, as can be seen in Figure 4, the sample CS3 contains a quartz phase, which may react with alumina to form mullite at 1050–1200 °C [46,47,48]: the amount of the quartz phase decreases and mullite appears. Starting from 1190–1200 °C, a sharp increase in volume begins, which is associated with the release of gases.

**Figure 10:**
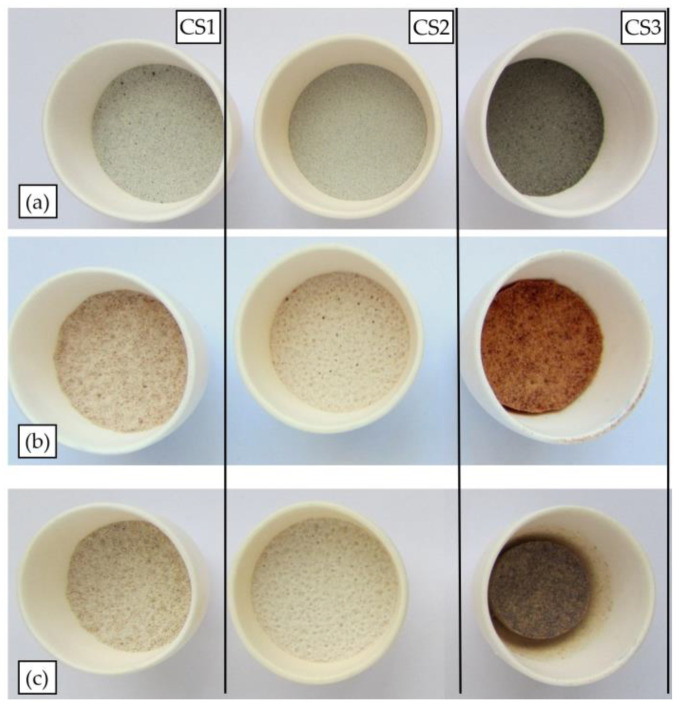
Images of cenospheres at various stages of heat treatment: (**a**) as-received, (**b**) 1100 °C and (**c**) 1200 °C.

As can be seen in Figure 10, heating to 1200 °C does not cause visually detectable changes for cenospheres CS1 and CS2, but a slight change in the colour of CS1 is observed, which can be explained by the burnout of coal particles and the oxidation of the Fe present in small quantities, which is black, to Fe_2_O_3_, which has a reddish-brown colour. In the case of sample CS3, a significant discolouration and compaction of the sample is observed at 1100 °C, and is even more pronounced at 1200 °C. The visually observed phenomena are consistent with the HTM experiment data.

### 3.7. Specific Thermal Conductivity Coefficient

Specific thermal conductivity is an important physical parameter for predicting the properties of CS-made composites. The thermal conductivities obtained from experiments are 0.109 ± 0.0005, 0.100 ± 0.0005, and 0.096 ± 0.0005 W·m^−1^·K^−1^ for CS1, CS2, and CS3. The data obtained on the thermal conductivity of CS allow us to conclude that if these particles are used as a filler in any matrix, a very low thermal conductivity can be achieved. This is primarily due to the fact that the freely-poured particles contain a lot of air, both internally (voids inside and pores in the walls) and between the particles.

The indicators that were determined during the research (chemical composition, thermal conductivity, bulk density) for samples CS1 and CS2 are rather similar, but for CS3, they differ somewhat. Sample CS3 differs from CS1 and CS2 considerably, with its thermal durability—these cenospheres sinter and shrink considerably. This may be due to the relatively greater number of particles with d of <63 µm, and a higher content of volatile and combustible carbon, at around 3.9 % (mass). It was also observed that a greater amount of the amorphous phase was present after heat treatment, with greater contents of SiO_2_.

## 4. Conclusions

The physical and chemical properties of microballoons of aluminosilicates, called cenospheres, which are produced as a by-product (a component of fly ash) of burning coal, were studied. Several sources of fly ash from coal basins in thermal power plants were selected. Cenospheres with particle sizes ranging from 40 to 500 μm were studied. Different particle distribution by size was observed, and the most uniform distribution of CS particles was in the case of CS2: above 74% with dimensions from 100 to 150 μm. The CS bulk had a similar density for all samples and amounted to around 0.4 g·cm^−3^, with a particle shell material density of 2.1 g·cm^−3^.

Energy-dispersive X-ray spectrometry (EDS) and chemical analysis of the CS revealed that the main components of the studied CS were SiO_2_ and Al_2_O_3._ In the case of CS1 and CS2, the sum of these components was on average from 93 to 95%. In the case of CS3, the sum of SiO_2_ and Al_2_O_3_ did not exceed 86%, and Fe_2_O_3_ and K_2_O were present in appreciable quantities in CS3.

The presence of SiO_2_ and Al_2_O_3_ in the composition of the microballoons was also confirmed by spectroscopic FTIR studies. As shown by X-ray studies, the diffractograms of the samples CS1 and CS2 were very similar; in the output samples, one can mainly observe the mullite Al_6_Si_2_O_13_ and the amorphous phase. In the output samples of CS3, mainly quartz and amorphous phases can be observed; the heat treatment caused a decrease in the quartz phase, and the crystalline phase of mullite appeared.

The thermal conductivity of all the CS samples varied from 0.096 to 0.109 W·m^−1^·K^−1^. The chemical composition, size distribution, and structure of the cenospheres affect their thermal stability. Cenospheres CS1 and CS2 did not sinter during heat treatment up to 1200 °C, while the sample CS3 was subjected to sintering already at 1100 °C, making it a bad choice for SF development. This behaviour in CS3 is attributed to the softening of the structure due to the presence of the quartz phase, Fe_2_O_3_ and K_2_O.

Due to the properties discussed above, CS1, which has similar thermal characteristics to sample CS2, should be selected for further experiments of matrix-less SF composite preparation or future scale-up, having better thermal stability, a narrow grading composition, and more uniform particle size distribution (94.2% of particles have a size of 63–150 µm). The morphology is also uniform, with high sphericity and a very low presence of agglomerates. Although there was a significantly higher percentage of defective particles in the as-received CS, the post-sieved product was of high quality.

## Figures and Tables

**Table 1:** Source designation and granulometric composition of CS.

Sample Designation	Coal Field	Grading Composition * (µm)
CS1	Donetsk	40–500
CS2	Donetsk	40–200
CS3	Ekibastuz	50–300

* according to the supplier data.

**Table 2:** Bulk density of CS, the density of the material, and interparticle void fraction.

Material	Bulk Density, g·cm^−3^	Pycnometric Density g·cm^−3^	Voids, %
Raw CS1	0.415 ± 0.004	2.153 ± 0.001	40.0
Raw CS2	0.380 ± 0.002	2.272 ± 0.001	43.0
Raw CS3	0.411 ± 0.004	2.301 ± 0.001	38.0
CS2 63–150 µm treated at 1100 °C	0.39 ± 0.004	2.185 ± 0.001	44.0
CS1 150–250 µm treated at 1100 °C	0.410 ± 0.004	2.178 ± 0.001	46.0
Ti@CS *	0.400 ± 0.004	2.661 ± 0.001	44.0
Ti-TiN@CS *	0.420 ± 0.004	2.531 ± 0.001	43.0

* CS2 63–150 µm treated at 1100 °C and then PVD coated.

**Table 3:** Average CS1, CS2, and CS3 element analysis in at. %.

Sample	C	O	Al	Si	Fe	Ca	Ti	Na	K	Mg	Si/Al*
CS1	10.04	55.48	14.92	17.37	0.31	0.25	0.29	0.23	-	0.02	1.16
CS2	8.94	57.16	15.25	16.76	0.24	0.04	0.33	-	-	-	1.10
CS3	11.49	53.00	8.50	21.00	2.02	0.05	0.40	-	2.0	0.74	2.47

Si/Al*—Si/Al at. ratio.

**Table 4:** Chemical composition of cenospheres in wt.%.

Sample	SiO_2_	Al_2_O_3_	Fe_2_O_3_	CaO	MgO	Na_2_O	K_2_O	LOI * 400 °C,%	LOI * 1000 °C,%
CS1	56.5	36.9	1.4	2.4	1.2	1.1	0.5	0.5	0.1
CS2	53.8	40.7	1.0	1.4	0.6	0.5	0.4	0.6	0.4
CS3	61.4	24.4	3.4	1.9	1.6	0.5	2.2	3.9	0.2

* LOI—Loss on ignition.

## Data Availability

Not applicable.
